# Three-dimensional volumetric evaluation of the different mandibular segments using CBCT in patients affected by juvenile idiopathic arthritis: a cross-sectional study

**DOI:** 10.1186/s40510-021-00380-6

**Published:** 2021-10-01

**Authors:** Davide Cavagnetto, Andrea Abate, Alberto Caprioglio, Paolo Cressoni, Cinzia Maspero

**Affiliations:** 1 Department of Biomedical Surgical and Dental Sciences, University of Milan, 20142 Milan, Italy; 2 Fondazione IRCCS Cà Granda, Ospedale Maggiore Policlinico, 20142 Milan, Italy

**Keywords:** Juvenile idiopathic arthritis, Temporomandibular joint, Cone-beam computed tomography, Facial asymmetry, Mandibular condyle

## Abstract

**Background:**

There is currently no information on how different mandibular segments are affected by juvenile idiopathic arthritis. The aim of this paper is to assess volumetric differences of different mandibular segments in subjects with unilateral and bilateral JIA and to compare them with non-JIA control volumes.

**Materials and methods:**

Forty subjects with unilateral TMJ involvement and 48 with bilateral TMJ involvement were selected for the case group and 45 subjects with no known rheumatic comorbidities for the control group. The mandible of each subject was divided according to a validated method into different paired volumes (hemimandible, condyle, ramus and hemibody).

**Results:**

The ANOVA test revealed a statistically significant difference in all the groups for condylar and ramus volumes, and the pairwise comparison evidenced a statistically significant higher condylar and ramus volume in the control group (1444.47 mm^3^; 5715.44 mm^3^) than in the affected side in the unilateral JIA group (929.46 mm^3^; 4776.31 mm^3^) and the bilateral JIA group (1068.54 mm^3^; 5715.44 mm^3^). Moreover, there was also a higher condylar volume in the unaffected side in the unilateral JIA group (1419.39 mm^3^; 5566.24 mm^3^) than in the bilateral JIA group and the affected side in the unilateral JIA group.

**Conclusions:**

The affected side of unilateral JIA patients showed statistically significant lower volumes in the hemimandible, in the condyle and in the ramus. The largest total mandibular volume was observed in the control group, followed by the unilateral JIA group and, lastly, by the bilateral JIA group.

## Background

Juvenile idiopathic arthritis (JIA) involves a group of conditions with joint inflammation (arthritis) with a female-to-male ratio of 3–6:1 [1]. It is the most common childhood rheumatic disease affecting children in Europe and North America and is one of the leading causes of acquired disability in children [2, 3]. It is a chronic condition that first appears before the age of 16 years, with idiopathic inflammation in at least one joint, and that lasts for at least 6 weeks [2].

The affected joints have classical signs of inflammation, i.e., joint pain, swelling, tenderness, stiffness, redness and warmth, a small, asymmetrical and hypoplastic mandible, a skeletal open bite, a short mandibular ramus, increased gonial angle and anterior facial convexity [4–6].

The temporomandibular joint (TMJ) is often affected by the rheumatic disease, with a clinically detectable prevalence that varies between 38 and 72% and depends on the diagnostic method applied and the JIA subtypes involved [7, 8].

The most active growth center for the mandible is located on the condylar head joint surface and, consequently, is often damaged by this rheumatic condition leading to an altered, asymmetrical growth [9, 10]. The reason for this growth impairment is poorly understood, and there are several factors that might contribute to this, such as the presence of high levels of proinflammatory cytokines and chronic use of corticosteroids (CS) to control JIA, malnutrition and immobilization [8].

It has been reported that chronic inflammation and progressive disruption of the condylar cartilage during mandible development are the main underlying factors involved in maxillomandibular growth anomalies. The mandibular ramus of affected condyles is shortened and often asymmetrical [11–13].

The condylar surface damage is frequently clinically silent, i.e., it evolves without subjective or clinical expressions, which often causes a delay in diagnosis, Moreover, reliable monitoring during therapy is often hampered by the absence of pain and palpable swelling [14, 15]. Symptoms, such as decreased mouth opening capacity (deviation of the mandible on mouth opening) and joint noises (crepitation and/or clicking), are not only usually noted late, but are also often underestimated [16]. This clarifies why advanced condylar lesions and severe malocclusion are such common findings in these patients [17]. An early diagnosis of TMJ arthritis is essential to prevent condylar destruction. At the time when clinical and morphological signs like mandibular retrusion or jaw asymmetry become obvious, the condyles are already irreversibly damaged [18].

The condylar head of JIA patients commonly shows signs of flattening and erosion to a different extent and gravity. How seriously the condylar head has been involved in the articular damage varies from small erosions and osteophytes to the complete absence of the condylar head and consequently different degrees of functional impairments [16, 19]. There are complex, intertwined underlying factors responsible for growth abnormalities, e.g., articular degenerative processes, age at TMJ involvement onset and the severity and activity of the rheumatic condition [20, 21].

Huntjens et al. and Koos B et al. carried out two similar studies on cone-beam computed tomography (CBCT) and reported that mandibular asymmetry in children with JIA is a consequence of significant alterations in the volume and morphology of the condylar head and the mandibular ramus [22, 23]. Cone-beam computed tomography (CBCT) and magnetic resonance imaging (MRI) are frequently used modalities for an early assessment of disease activity in JIA and the follow-up of these patients [24]. MRI is considered the best available examination test for determining TMJ active inflammatory process and soft tissue pathological alterations [25, 26].

The aim of TMJ imaging is to make a precise assessment of cortical and trabecular bony structure involvement and to evaluate disease extent and progression. The aim of TMJ imaging is to assess the cortical and trabecular bony structure involvement and to evaluate disease extent and progression. TMJ degenerative arthritis is usually not detectable on panoramic x-ray until an advanced articular involvement stage, when bony lesions are present and destructive abnormalities of condylar heads have already begun [27]. Structure superimposition in bi-dimensional radiographs hampers TMJ evaluation [28]. CBCT allows for early diagnosis of degenerative joint disease as it has a higher sensitivity in the detection of small bony structure alterations than does conventional radiology and MRI, which is indeed more useful for rapid control of active synovitis and prevention of structural damage [22]. CBCT may also identify asymptomatic articular involvement in certain cases before any significant impairment has taken place in the maxillofacial growth pattern [17].

CBCT volume imaging generates high-resolution multiplanar reconstruction images with short scanning times and lower radiation doses than multi-slice computed tomography (MSCT). It provides an accurate representation of the temporomandibular joint and joint space and anatomical structures without the typical distortional projection typical of conventional radiology [29, 30]. That is why, CBCT is the diagnostic radiographic examination of choice for the assessment of TMJ bony changes [31, 32].

The primary aims of this paper are: to assess whether there are volumetric differences in different mandibular components between the affected and unaffected sides in JIA patients with unilateral TMJ involvement and to identify any differences between JIA patients with unilateral TMJ and those with bilateral TMJ involvement and healthy subjects so as to determine which mandibular segment is mostly affected.

## Materials and methods

### Study design

This retrospective cross-sectional study was performed on the CBCTs of juveniles affected by JIA and those of subjects without a JIA diagnosis as controls. The patients’ parents gave written informed consent to carry out an anonymous analysis of their children’s medical history for research purposes. The study protocol was approved by the Ethical Committee of the Fondazione IRCCS Cà Granda, Ospedale Maggiore, Milan, Italy (protocol no. 573/15). This study was performed in compliance with the Declaration of Helsinki for human studies.

### Types of participants and inclusion criteria

The clinical records of JIA patients, treated between April 2012 and May 2017, were retrieved from the Department of Biomedical Surgical and Dental Sciences at the University of Milan, Fondazione IRCCS Cà Granda, Ospedale Maggiore Policlinico, Milan. The CBCT scans of the patients including at least mandibular were extrapolated from the archives of the Department of Biomedical Surgical and Dental Sciences, University of Milan, Italy.

The information gathered from the JIA patients’ medical history was: gender and medical records, age at first reported occurrence of JIA manifestations and unilateral or bilateral affection of the temporomandibular joint.

**Fig. 1:**
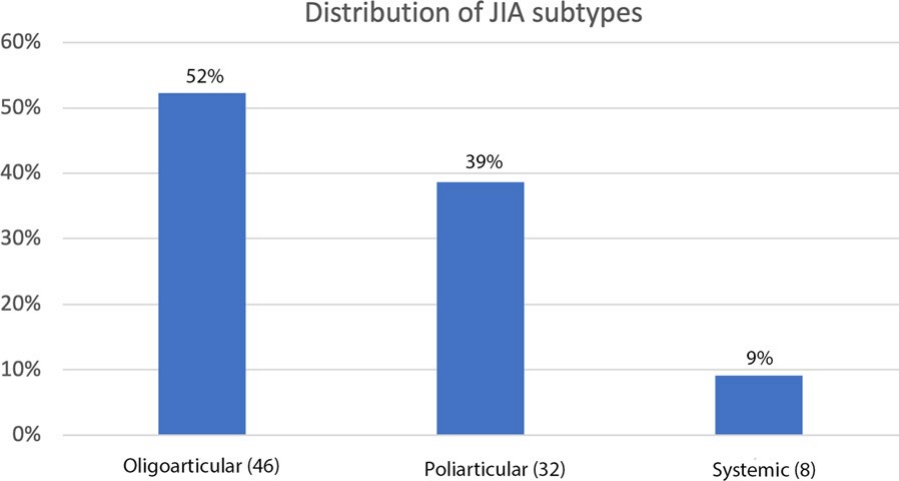
Distribution of JIA subtypes in the present study according to ILAR criteria

Inclusion criteria for JIA patients with were: diagnostic classification according to the International League of Associations for Rheumatology criteria (ILAR) [33] reported in patients’ records (Fig. 1); TMJ involvement; a good quality CBCT scan of the temporomandibular joint; no history of craniofacial trauma or any other alterations in craniofacial growth due to an underlying syndrome; no history of orthopedic or orthodontic treatment; and no congenital birth defect involving the craniofacial area.

Inclusion criteria for controls, aimed at obtaining a clinically meaningful comparison, were: physiological anatomy of the condylar head and symmetric mandibular growth; gender and age matching with the JIA group; skeletal class II, according to Riedel’s cephalometry [34] (ANB > 4°); subjects with an increased intermaxillary divergence (Pns-Ans^Go-Me > 37°); no congenital birth defect; genetic or trauma-induced craniofacial anomalies; no family history of rheumatic diseases; and no history of maxillofacial orthopedic surgery or orthodontic treatment.

Eighty-eight Caucasian subjects, 74 females and 14 males (average age 11.6 ± 4.3), with a JIA diagnosis and temporomandibular joint involvement were selected into the study. All JIA subjects had clear radiological signs of articular degenerative changes (flattening or erosion) in at least one TMJ. Forty subjects (34 females and 6 males) had unilateral TMJ involvement (average age 12.3 ± 4.6), and 48 (40 females and 8 males) had bilateral TMJ involvement (average age 11.3 ± 4.7 years). All these patients presented the classical craniofacial characteristics of JIA patients with TMJ involvement, that is an underdeveloped mandible, resulting in a skeletal class II according to Steiner and an increased maxillomandibular divergence [35].

Forty-five Caucasian Italian subjects with no known rheumatic comorbidities, 36 females and 9 males (average age 11.5 ± 4.4), were randomly selected as the first 45 to match our criteria of inclusion in the department archive and then included into the control group. Cone-beam CT scans were obtained from the aforementioned department. The cone-beam CT scans had been done for various reasons, e.g., (a) a complex tooth extraction (e.g., wisdom teeth, supernumerary teeth, etc.); (b) assessment of retained teeth; (c) assessment of odontogenic or non-odontogenic jaw cysts; (d) assessment of ear, nose and throat (ENT)-related upper respiratory tract disturbances, e.g., sinusitis, odontogenic and non-odontogenic maxillary sinus cysts); and (e) virtual planning of temporary anchorage devices (TAD) installation.

### CBCT examination and postprocessing

All the cone-beam CT (CBCT) scans were taken by a cone-beam i-CAT FLX unit (Imaging Sciences International, Inc., https://ct-dent.co.uk/i-cat-vision/). The machine was set for full rotation, at 300 image frames, at 120 kVp, 5 mA with a pulsed exposure time of 3.7 s, a voxel size of 0.4 mm and fields of view (FOV) of 16 × 8 or 16 × 11 mm. The CBCT scans were saved as DICOM files (Digital Imaging and Communications in Medicine) that is the international standard to transmit, store and process medical imaging. Volumetric rendering of DICOM files, segmentation and analysis of mandibular corpus, ramus and condylar head was performed by Mimics Research ™ v.20 software (NV, Technologielaan 15, 3001 Leuven, Belgium, https://www.materialise.com/en/medical/mimics-innovation-suite/mimics). All measurements were made by an orthodontist (A.A) experienced in 3D dental imaging.

**Fig. 2:**
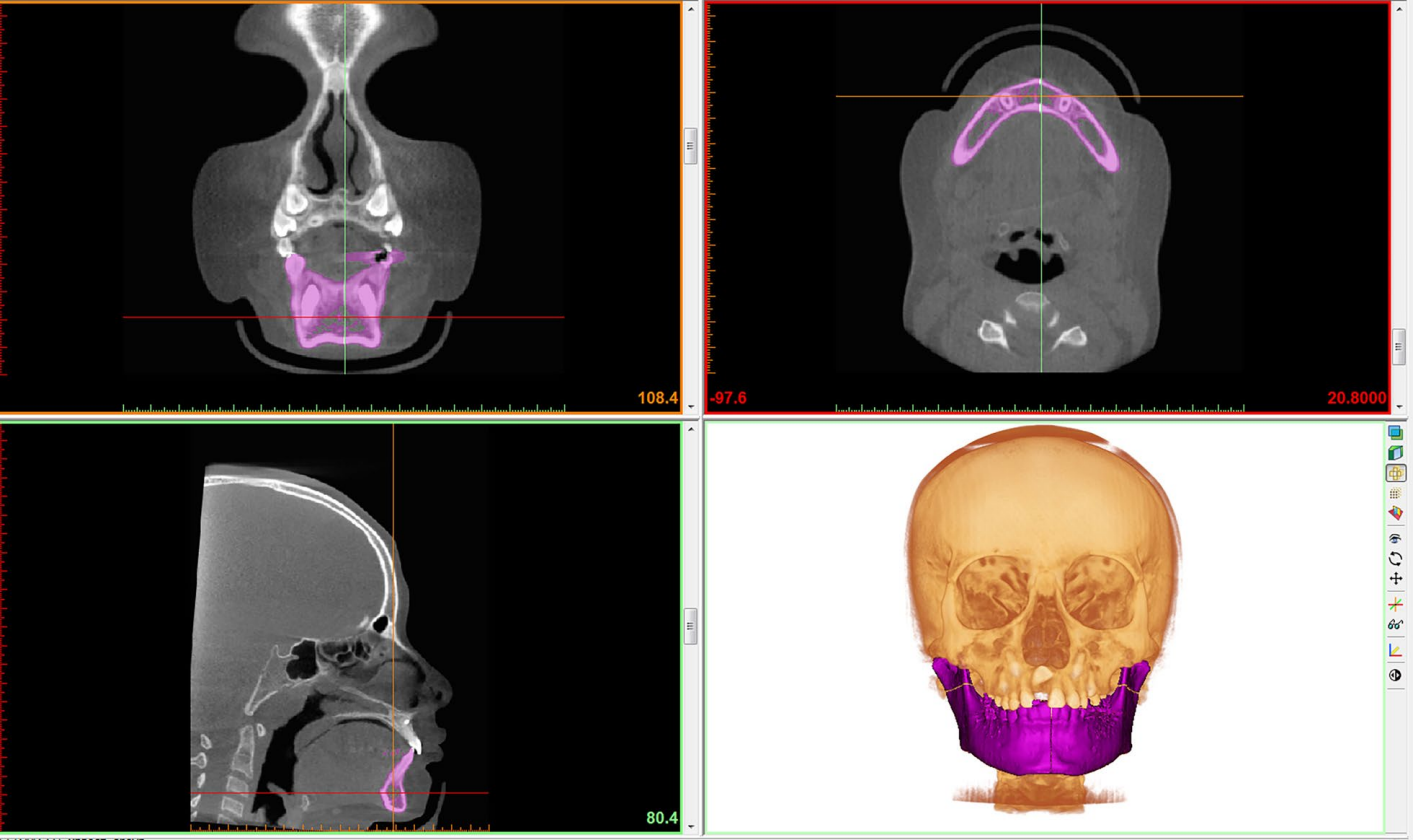
Software view of the first step of mandibular segmentation, that is the definition of whole mandibular volume using Materialise™ Mimics software

A segmentation mask was created to obtain volumetric reconstruction of the mandibular parts of interest. The whole mandibular bony structure was isolated in the first mask (Fig. 2) using the threshold option set by Mimics Research™ software.

**Fig. 3:**
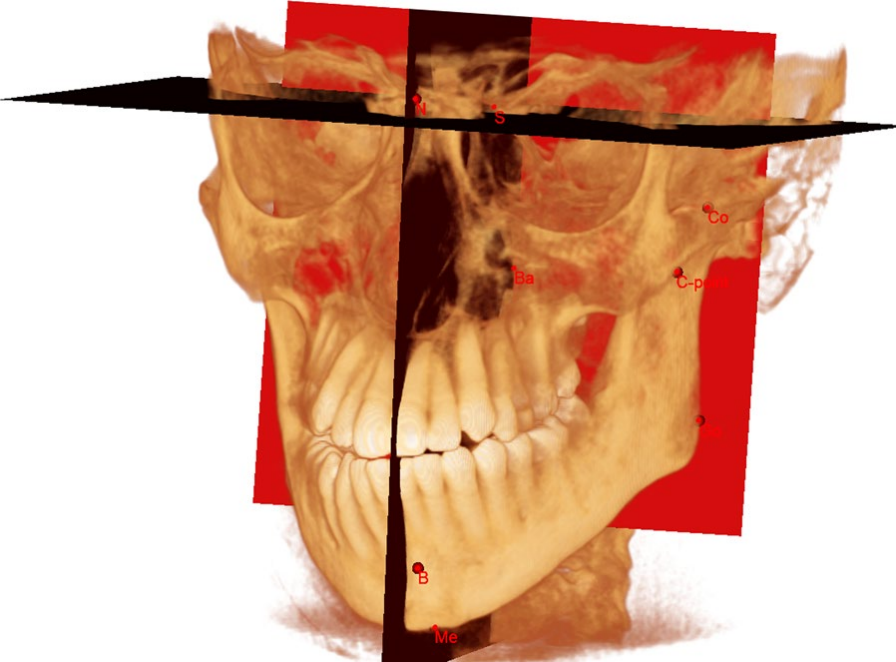
Software view of the cephalometric reference system used in the present study

This mask was then divided into the anatomical components of the mandibular bone using a method where reliability and reproducibility have already been validated by Nolte et al. [36] A 3D cephalometric reference system was identified, as described by Swennen et al. [37], and the horizontal (x) plane was defined as the one passing through Sella (S) obtained tilting six degrees below the plane, passing through Sella and Nasion (N) and normal to the mid-sagittal plane (S–N–Ba). The vertical reference plane (y) was defined as the one passing through S and normal to *x*. The sagittal reference plane (z) was defined as the one passing through S and normal to x and y (Fig. 3).

The following points for both the right and left mandibular side were identified: Condylion (the most superior posterior point on the condylar head), Gonion (the most posterior inferior point on the angle of the mandible), the C-point (defined as the lowest point of the sigmoid notch), Down’s B point (the rearmost point on the external contour of the mandibular symphysis), Menton (the lowermost point on the mandibular symphysis) and mental spine point (the center of the four genial tubercles of the mandible) [38].

**Fig. 4:**
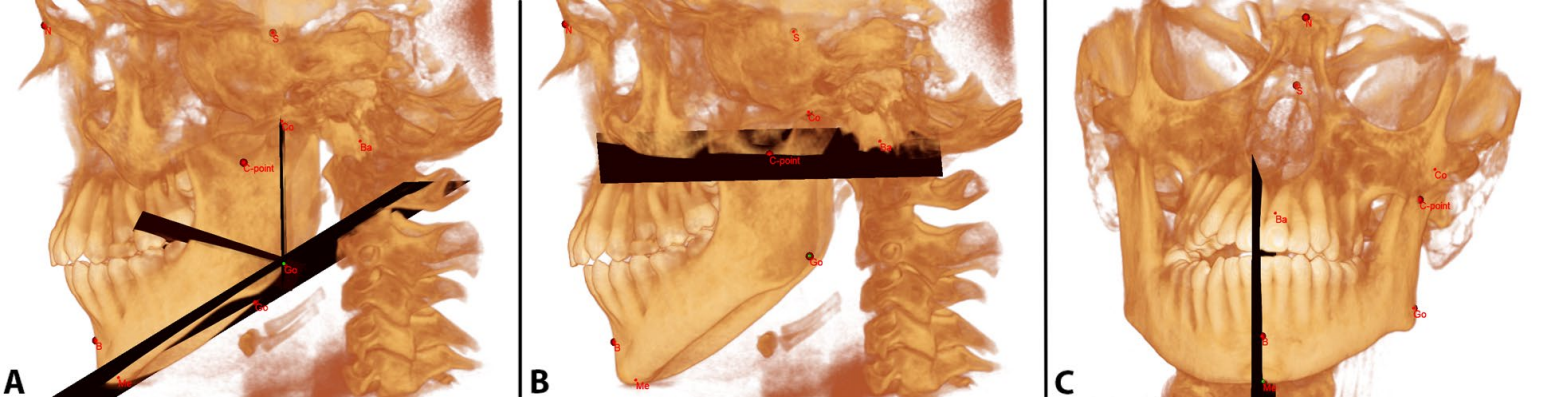
Additional mandibular planes used in the current study: **A** Condylion–Gonion plane, Gonion–Menton plane and Mandibular Angle plane; **B** C-point plane; **C** Median plane

The following planes were identified: (1) the Condylion–Gonion plane, i.e., the one passing through Condylion and Gonion, normal to the aforementioned reference plane according to Swennen; (2) the C-point plane, passing through the C-point, parallel to the horizontal plane of the reference frame; (3) the Gonion–Menton plane, passing through the left and right Gonion and Menton; (4) the Median plane, passing through Menton, Down’s B point and the mental spine point; (5) the Mandibular Angle plane, i.e., the bisecting plane between planes 1 and 2 (Fig. 4).

A stepwise approach was used to define the volumes of the anatomical components of the mandible from each mandibular 3D mask. The mandibular mask was then isolated from the rest of the maxillofacial skeletal structures. After separation, a 3D volumetric structure of the mandible was produced. The mandibular canal was then flood-filled and all the teeth were eliminated from the 3D mask so as to consider only the mandible bony structures. To the best of our knowledge, the only currently scientifically validated method to do so is the one described by Nolte et al. [36]; consequently, it was adopted in the present study. The emergence profile from the alveolus of each tooth was marked on the software for every patient, and a tailored cutting plane was defined. This procedure allowed for the removal of the clinical crowns of each tooth from the mandible volume.

**Fig. 5:**
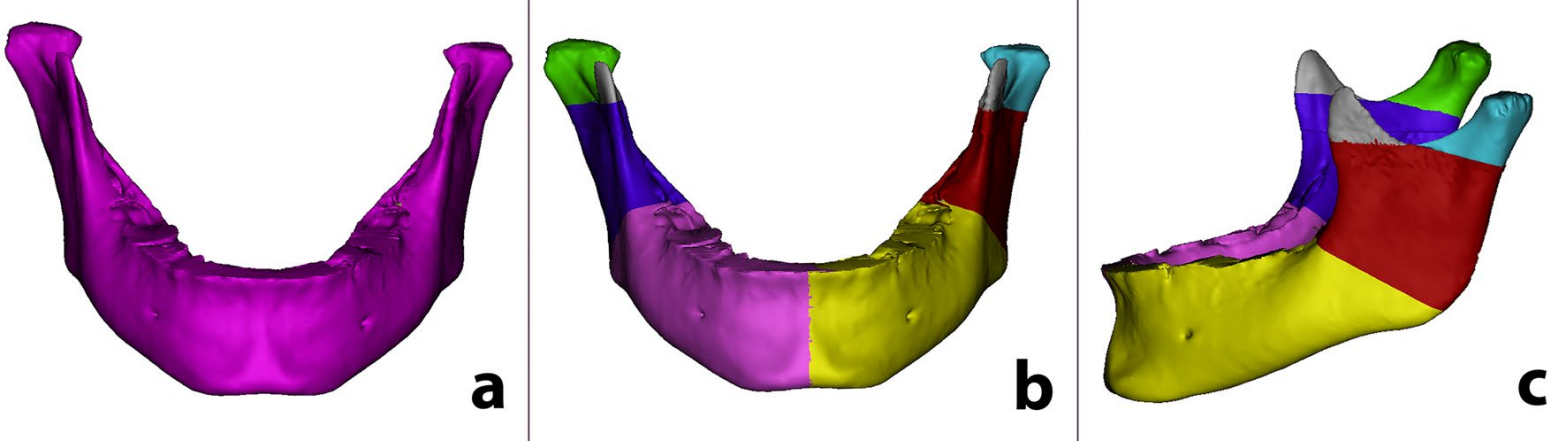
Example of the steps taken to reconstruct the volumes that have been considered in this study in a patient affected by unilateral TMJ involvement. **A** The whole mandibular volume has the clinical crowns removed from each tooth. It is performed after the whole mandible has been isolated to remove all biases connected to clinical crown sizes. **B** The mandibular volume is split into two hemi-mandibular volumes. **C** In each hemimandible, the following volumes have been completed: corpus, ramus and condyle

After that, the volumes under investigation could be segmented and computed as follows: the condyle: the volume defined as the one above the C-point plane not considering the coronoid process as it does not belong either to the condyle or the ramus; the ramus: the volume between the C-point plane and the Mandibular Angle plane; and the hemibody: the volume between the Mandibular Angle plane and the Median Landmark plane (Fig. 5). As the coronoid process is not particularly affected by JIA, it was not taken into consideration.

The flood-fill algorithm was processed by Mimics Research™ software to calculate the whole mandibular volume excluding trabecular porosity and scattering. Lastly, Mimics Research™ software calculated the predefined volumes automatically.

### Statistical analysis and sample size calculation

A priori sample size calculation was performed with G*Power (version 3.1.9, http://www.psychologie.hhu.de/arbeitsgruppen/allgemeine-psychologie-und-arbeitspsychologie/gpower.html). Mean and standard deviation of condylar volume of affected condyles (1007.82 ± 384.27) and not affected condyles (1424.69 ± 417.64) reported by Farronato et al. [39] were taken as reference values. On this basis, 23 subjects per group were computed to be needed to reject the null hypothesis that the population means of the case and control groups are equal with probability (power) 0.95. The type I error probability associated with this test of this null hypothesis is 0.05.

Statistical analysis was performed with SPSS v21.0 (version 25.00; IBM Corp, Armonk, NY, https://www.ibm.com/support/pages/release-notes-ibm®-spss®-statistics-250).

Subjects with JIA were divided according unilateral or bilateral damage to the temporomandibular joints.

Dimensional variations of the total mandible, hemimandible, condyle, ramus and body were analyzed and compared with healthy control group. The compatibility assessment between the JIA and non-JIA groups was performed in relation to age, ANB angle, intermaxillary divergence using an independent t-test and in relation to gender using a Chi-squared test.

Gaussian distribution of data was assessed using Shapiro–Wilk test. Since data were normally distributed, variables were reported as mean and standard deviation. Two-tailed independent t test was used to compare volumes of each variable between: affected and non-affected sides in unilateral JIA subjects; right and left sides in bilateral JIA patients; and right and left sides in non-JIA control group. One-way analysis of variance (ANOVA) with the post hoc Tukey’s test with Bonferroni’s correction was used to assess if difference in volume between unilateral affected side in unilateral JIA patients, unilateral unaffected side in unilateral JIA patients, bilateral JIA patients and non-JIA healthy group was significantly different for all the variables considered. For bilateral JIA patients and healthy control groups, one-way analysis of variance (ANOVA) was conducted using the mean and standard deviation between right and left sides, as no significant difference has been found between the two sides. Instead, for unilateral JIA subjects’ each mandibular segment has been evaluated separately. Percentage difference of volume between mandibular parts has been calculated.

Significance level for both ANOVA and two-tailed independent *t* test was set at 0.05.

### Method error

The intra-class correlation coefficient (ICC) test was adopted to evaluate intra-operator and inter-operator reliability, while the Dahlberg’s formula to measure the method error. All the cone-beam scans were acquired by the same experienced dental radiologist and the first set of measurements were taken by researcher A.A. Two weeks later, 20 cone-beam CT scans were extrapolated at random and re-evaluated by a different researcher D.C and then the same CBCT scans were evaluated again by researcher A.A to assess intra-observer and inter-observer reliability. The researchers were blinded to the patients’ identity.

## Result

The compatibility assessment between the JIA and non-JIA groups retrieved no statistically significant differences, thus reducing biases when comparing the groups (Table 1). Descriptive statistics and statistical comparisons for each mandibular volumetric measurement between the affected and unaffected sides in unilateral JIA patients and between the right and left sides in bilateral JIA patients and the control group are reported in Table 2. There was a statistically significant difference between the affected and unaffected sides for each variable considered in the unilateral JIA group (Table 2) with greater values in the unaffected side, whereas no statistically significant difference was observed in either the bilateral JIA group or the control group between the right and left sides for each variable (Table 2).

**Table 1:** Demography and clinical characteristics of the patients with the relative statistical analysis calculated by means of *t* test for the age group, ANB angle and intermaxillary divergence comparison and Chi-squared test for differences in proportion

Sample characteristics	Total (*N* = 133)	JIA group (*N* = 88)	Control group (*N* = 45)	Significance (*p* value)
Mean age ± standard deviation	11.55 ± 4.13	11.6 ± 4.3	11.5 ± 4.4	0.434
**Sex**				
Male	23	14	9	0.082
Female	110	74	36	
**Skeletal class**				
ANB angle	6.82 ± 2.1	7.05 ± 2.2	6.58 ± 1.7	0.173
Intermaxillary divergence	37.6 ± 1.9	38.4 ± 2.6	36.8 ± 1.5	0.351

*p value < 0.05 was considered as statistically significant

**Table 2:** Descriptive statistics and two-tailed independent T test between affected (Uni-Aff) and unaffected (Uni-UnAff) sides in Unilateral JIA patients and between right and left sides in bilateral JIA patients (Bil-Aff) and control group (Ctrl)

Volume (mm^3^)	Unilateral JIA (*N* = 29)		Bilateral JIA (*N* = 48)				Ctrl (*N* = 25)				*p* value (UniAff-UnAff)	*p* value (Bil-Aff Right vs Left)	*p* valve (Ctrl Right vs Left)
	Uni-Aff	Uni-UnAff	Bil-Aff-Right		Bil-Aff-Left		Right		Left				
	Mean ± SD	Mean ± SD	Mean	SD	Mean	SD	Mean	SD	Mean	SD	–	–	–
Hemimandible	22,441.74 ± 4964.13	24,815.70 ± 5148.13	22,749.07	4781.48	22,591.13	4807.11	24,476.92	4739.22	25,200.76	4888.02	0.021*	0.87	0.72
Condyle	929.46 ± 261.88	1419.39 ± 375.23	1076.4	425.08	1061.04	406.44	1381.481	181.32	1482.26	180.09	0.0084*	0.85	0.094
Ramus	4776.31 ± 1360.85	556 6.24 ± 1541.79	4953.29	1505.22	4872.06	1390.18	5764.82	1464.51	5653.41	1454.32	0.033*	0.78	0.81
Hemibody	16,674.71 ± 3136.04	17,895.79 ± 3094.57	16,738.46	3081.41	16,579.14	3169.58	17,737.88	15,521.89	17,921.89	2946.14	0.078*	0.92	0.68

*p value < 0.05 was considered as statistically significant

Descriptive statistics and statistical comparison between the unilateral affected side, the unilateral unaffected side, the bilateral JIA group and the control group are reported in Table 3. As there was no statistically significant difference between the affected sides in the bilateral JIA group and the control group, the mean and standard deviation of both sides were adopted for the statistical comparison.

**Table 3:** Descriptive statistics and statistical comparison with one-way analysis of variance between the following groups: unilateral affected side (Uni-Aff), unilateral unaffected side (Uni-UnAff), bilateral JIA patients (Bil-Aff) and control healthy group (Ctrl)

Volume (mm^3^)	Uni-Aff (*N* = 29)	Uni-UnAff (*N* = 29)	Bil-Aff ^A^ (*N* = 48)	Ctrl^A^ (*N* = 25)	ANOVA test	Pairwise comparisons (Bonferroni correction)
	Mean ± SD	Mean ± SD	Mean ± SD	Mean ± SD	*P* value	
Hemimandible	22,441.74 ± 4964.13	24,815.70 ± 5148.13	22,670.10 ± 4783.78	25,007.70 ± 4635.16	0.057	–
Condyle	929.46 ± 261.88	1419.39 ± 375.23	1068.54 ± 410.2	1444.47 ± 170.82	0.0012*	Ctrl > Uni-Aff < Uni-UnAff; Ctrl > Bil-Aff < Uni-UnAff
Ramus	4776.31 ± 1360.85	5566.24 ± 1541.79	4812.67 ± 1425.15	5715.44 ± 1407.63	0.037*	Ctrl > Uni-Aff < Uni-UnAff; Ctrl > Bil-Aff < Uni-UnAff
Hemibody	16,674.71 ± 3136.04	17,895.79 ± 3094.57	16,688.80 ± 3109.74	17,847.57 ± 2766.12	0.201	–
Mandible Tot ^a^	47,256 ± 5086.37^B^		45,340.76 ± 4787.21	49,676.92 ± 4783.38	0.0018*	(Uni-Aff-UnAff) < Ctrl; Bil-Aff < Ctrl

*Uni-Aff* affected side—unilateral JIA, *Uni-UnAff* unaffected side—unilateral JIA, *Bil-Aff* bilateral JIA, *Ctrl* control group

*p value < 0.05 was considered as statistically significant

^A^Values are means ± standard deviations of each patient average value between right hemimandible and left hemimandible

^B^Values are means ± standard deviations of each patient average value between unilateral affected side and unilateral unaffected side

No statistically significant difference was observed in the hemimandible and hemibody volume between the groups (Table 3).

There was a statistically significant difference in all the groups for condylar and ramus volumes, and the pairwise comparison evidenced a statistically significant higher condylar and ramus volume in the control group (1444.47 mm^3^; 5715.44 mm^3^) than in the affected side in the unilateral JIA group (929.46 mm^3^; 4776.31 mm^3^) and the bilateral JIA group (1068.54 mm^3^; 5715.44 mm^3^). Moreover, there was also a higher condylar volume in the unaffected side in the unilateral JIA group (1419.39 mm^3^; 5566.24 mm^3^) than in the bilateral JIA group and the affected side in the unilateral JIA group.

There was a statistically significant difference in mandibular total volume between the JIA groups (both unilateral and bilateral) and the control group. The pairwise comparison evidenced the highest mandibular total volume in the control group (49,676.92 mm^3^), followed by the unilateral JIA mandible (47,256 ± 5086.37 mm^3^) and then the bilateral JIA mandible (45,340.76 mm^3^) (Table 3).

Table 4 summarizes the mean differences with the corresponding percentage difference in terms of volume between mandibular units.

**Table 4:** Mean difference, standard deviation (SD) and percentage (%) difference of volume for each variable between the following groups: unilateral affected side (Uni-Aff), unilateral unaffected side (Uni-UnAff), bilateral JIA patients (Bil-Aff) and control healthy group (Ctrl)

Volume (mm^3^)	∆ (Uni-UnAff **−** Uni-Aff)	Relative difference	∆ (Uni-Aff **−** Bil-Aff ^A^)	Relative difference	∆ (Ctrl^A^ **−** Uni-Aff)	Relative difference	∆ (Uni-UnAff **−** BilAff)	Relative difference	∆ (Uni-UnAff ** −** Ctrl^A^)	Relative difference	∆ (Ctrl^A^ **−** BilAff)	Relative decrease
	Mean ± SD	%	∆Mean ± SD	%	Mean ± SD	%	Mean ± SD	%	Mean ± SD	%	Mean ± SD	%
Hemimandible	2741.63 ± 1672.81	10.1	− 228.36 ± 6001.08	1.2	2565.96 ± 8347.69	11.1	2145.6 ± 6412.55	8.9	− 192.00 ± 8185.45	0.8	2565.96 ± 7621.32	9.6
Condyle	510.59 ± 223.99	34.6	− 139.08 ± 529.45	12.7	515.01 ± 386.67	35.7	350.85 ± 630.01	25.3	− 25.08 ± 496.62	1.8	375.93 ± 390.98	26.2
Ramus	813.21 ± 398.47	15.2	− 136.36 ± 1945.89	0.8	939.13 ± 2511.52	16.5	753.57 ± 2074.63	13.4	− 149.20 ± 2574.916	2.7	902.77 ± 1969.66	16
Hemibody	1488.42 ± 1270.53	6.8	14.09 ± 3761.91	0.1	1172.86 ± 4837.85	6.6	1206.99 ± 3912.12	6.7	48.22 ± 4673.04	0.3	1158,77 ± 4681.54	6.5

	∆ (Unilateral JIA^B^ **−** Bilateral JIA^A^)				∆ (Unilateral JIA^B^ **−** Ctrl^A^)				∆ (Bilateral JIA^A^ **−** Ctrl ^A^)			
Mandible Tot ^a^	1916.24 ± 1137.49			4.1	− 2420.92 ± 3132.36			4.9	− 4336.16 ± 2357.68			9.8

*Uni-Aff* affected side—unilateral JIA, *Uni-UnAff* unaffected side—unilateral JIA, *Bil-Aff* bilateral JIA, *Ctrl* control group

*p value < 0.05 was considered as statistically significant

^A^Values are means ± standard deviations of each patient average value between right hemimandible and left hemimandible

^B^Values are means ± standard deviations of each patient average value between unilateral affected side and unilateral unaffected side

The ICC values for the intra-observer and inter-observer reliability showed high agreement for all the volumetric measurements evaluated; average (± SD, range) intra-observer and inter-observer ICC values were 0.975 (± 0.011, 0.964–0.991) and 0.968 (± 0.013, 0.954–0.987), respectively (Table 5). According to Dahlberg’s formula, the random error for mandibular volumetric measurements was 427 mm^3^ for the hemimandible, 152 mm^3^ for the condyle, 238 mm^3^ for the ramus and about 346 mm^3^ for the mandibular body. Overall, the method error was considered negligible.

**Table 5:** Intra-operator and inter-operator agreement for mandible segmentation

	ICC (intra-operator)	ICC (inter-operator)
Hemimandible	0.967	0.954
Condyle	0.983	0.978
Ramus	0.964	0.959
Hemibody	0.971	0.963
Mandible Tot^a^	0.991	0.987

*R* right, *L* left, *SD* standard deviation

^a^Sum between right hemimandible and left hemimandible

## Discussion

The main growth center of the mandible is located in the condyle and only a thin layer of fibrocartilage separates it from the joint space, making mandibular growth vulnerable to arthritic changes. Degenerative joint disease at the level of the temporomandibular joint in JIA patients may occur in only one (unilateral) or both (bilateral) TMJs. However, it usually starts in one, and with progression, in some time it may involve the other. This condition may cause mandibular asymmetry due to the different degree of progression in articular damage and varying growth impairments between the condyles [6, 22, 40–42].

Sagittal mandibular body growth is the result of bone resorption on the anterior border of the mandibular body and bone deposition on its posterior border [29]. The vertical growth of the ramus is a consequence of the growth within the condylar process [29, 43].

Asymmetrical involvement of the temporomandibular joints during growth (i.e., when only one is affected or when there is a significant difference in disease progression between the TMJs) may be associated with asymmetrical growth of the right and left mandibular *rami*, leading to ramus shortening and condylar volume reduction on the damaged side [44]. Patients with active TMJ damage associated with JIA during growth show a progressively worsening craniofacial morphology: posterior face height goes through a decelerated vertical development and might even be reduced during growth due to articular damage, skeletal and dental open bite and anterior crossbite might also occur.

Kjellberg et al. [45] were the first to report TMJ alterations in JIA patients. They measured condylar height on OPG [27] and noted that subjects suffering from JIA with TMJ involvement had shorter, and often asymmetrical, condylar *rami*. The advent of low-dose three-dimensional (3D) imaging techniques, i.e., CBCT, has allowed for a precise monitoring of the effects of disease progression and/or treatment (i.e., distraction splints, Andresen activators, etc.), enhancing understanding of this condition [39, 46, 47]. It has led to the possibility of assessing the condylar morphology and volume of affected and unaffected condyles, a comparison with non-JIA condyles and the monitoring of condylar changes with age [48, 49].

Huntjens et al. (2008) evaluated asymmetry in the condylar volumes of young JIA patients and reported significant asymmetry [22]. These findings were later confirmed in a similar study by Garagiola et al. [17].

Farronato et al. (2020) reported a statistically significant difference between the volumes of affected and unaffected JIA condyles and between affected condyles and non-JIA controls [39]. They stated that condylar volume is statistically significantly larger in healthy subjects than in unilateral or bilateral JIA patients, in line with what was previously reported by Demant et al. [50]. Moreover, they evaluated the head and neck volumes of the affected condyles and reported that they were both significantly smaller than unaffected condyles in patients with unilateral JIA and compared to healthy controls.

In the present study, a statistically significant smaller volume has been noticed comparing condyles, ramus and hemimandible of the affected side with the unaffected ones in unilateral JIA patients (Table 2).

Several authors [4, 20, 39] reported that ramus growth deficiency is the most commonly associated maxillofacial factor with the disease and the ramus is shorter in JIA patients than unaffected individuals. Although the present data on unilateral JIA subjects as to ramus deficiency were in line with those previously published in the literature, as 3D volumetric reconstruction was used, this research obtained more accurate information than that reported in other research that used a linear measurement (Table 2).

There were no statistically significant differences for subjects with bilateral JIA and non-JIA patients between the right and left sides of the TMJ for all the variables taken into consideration (Table 2). This observation evidenced symmetry, in terms of volume, in patients affected by bilateral JIA, as there was a symmetrical deficiency of each anatomical mandibular component.

The volume of the hemibody confirmed the authors’ expectations, i.e., there was no statistically significant volumetric difference between the two sides of the TMJ in bilateral or unilateral JIA and healthy subjects (Table 2). These results confirm that the mandibular body is only slightly or nothing affected, if at all, by this rheumatic condition.

Juvenile idiopathic arthritis has well-known effects on facial growth [11]. Indeed, JIA sufferers have impaired mandibular growth and a progressive clockwise rotation of the mandible [51]. Larheim and Haanes [52] and Stabrun [53] stated that there is a combination of factors involved in the impairment of mandibular growth including: pain, tenderness, existing malocclusion and reduced range of motion, all of which lead to a reduced growth *stimulus* as stated by Pedersen et al. [54]. In fact, a reduction in muscular function has been tested in vivo to reduce bone apposition rate because of a lower functional strain on the bone and less tension on the periosteum [55].

Predictably, there was a statistically significant difference among the groups in the condylar volume (Table 3). The non-JIA group and the unaffected side of unilateral JIA patients had bigger condyles than the bilateral JIA group and the affected side of unilateral JIA subjects, confirming the findings reported in the literature. There was an average difference in volume of 435 mm^3^, ranging from 25.3 to 35.7% between affected and unaffected condyles (Table 4).

Gonzalez et al. (2016) evaluated the 3D mandibular skeletal changes in JIA patients treated with a distraction splint and observed an asymmetry in condylar volume, vertical displacement and that the ramus of the affected side was not so long as the unaffected side [20].

Stoustrup PB et al. (2018) have recently studied the association between the types of radiologic TMJ abnormalities and facial development in JIA patients with TMJ involvement [56]. They reported that mandibular asymmetry was exclusively related to a short condyle on the affected side, while no significant differences were observed for ramus height, postulating that a shorter condyle was responsible for the decreased posterior face height in JIA patients. This was in contrast to data published by Farronato et al. [39], as they reported a significant shorter ramus height in patients affected by JIA. In contrast to Stoustrup et al. [56], the research carried out in this study evidenced a statistically significant difference among the groups at the level of the ramus (Table 3). This difference is allegedly due to the different method of measure. In fact, Stoustrup et al. [55] performed linear measurements over CBCT scans, while the present study adopted a three-dimensional approach to evaluate asymmetry. Noteworthy is the fact that a there was a clinically relevant volumetric difference of approximately 900 mm^3^ with an average of 15% volumetric difference between the affected and unaffected ramus, as detailed in Table 4. The ramus volume measurements obtained validated the considerable dimensional deficit of the affected mandibular morphology. Indeed, signs of absolute volumetric decrease in the mandibular ramus were observed for the pathological side.

When dealing with mandibular asymmetry, most articles reported on the vertical dimension of the condyle and ramus measured on panoramic radiographs of healthy subjects [57]. The side differences observed in condylar height in growing individuals may indicate that the functional forces on the TMJs and mandibular regions are not necessarily the same, leading to unequal growth of the condyle and ramus heights on the right and left sides. Turp et al. (1998) invested into the degree of mandibular asymmetry of 25 dry skulls, showing relative absolute differences between the right and left sides, attributed to biological individual variation [58].

Farronato et al. [39] reported that the asymmetry in JIA patients is more related to functional adaptations rather than to an altered condyle condition. In agreement with the present findings, Piancino et al. (2015) reported a significant difference between the condyles of JIA patients and a healthy control group. They also reported major asymmetry differences between females and males. However, in contrast to data obtained in this study, the authors reported no differences in the range of ramus asymmetry between their groups [59].

Koos et al. [23] and Huntjens et al. [22] investigated mandibular asymmetries by CBCT, confirming a significantly more pronounced asymmetries in the JIA group and a greater standard deviation in ramus length, even if no comparison was made with a healthy control group.

The authors are of the opinion that, given the importance of a better understanding of the underlying craniomaxillofacial factors involved in JIA, further studies should be carried out to validate and provide more information on this invalidating disease. The mandibular growth anomalies evidence the importance of an early diagnosis and the need for an early orthopedic–orthodontic therapy so as to limit growth disorders, which could complicate the proper development of the patient's craniofacial structures.

### Limitations and strengths of the study

Albeit the sample is sufficient from a statistical point of view, the authors are aware that it would be useful to evaluate the same parameters in a larger sample of subjects with JIA and assess whether dividing the main sample into different groups (i.e., considering the number of joints involved and sex) any difference emerges.

Moreover, the present research did not assess differences based on the type of JIA and the different pattern of TMJ involvement.

Furthermore, a study with a longitudinal design on condylar growth would provide further important data. However, the current state of diagnostic techniques would impede this, due to the need for repeated CBCT analysis over time. Therefore, the authors hope in further evolutions in the field of diagnostics so as to be able to perform volumetric analysis of the condyle minimizing exposure risks. The constant development of radiation-free imaging like magnetic resonance imaging (MRI) will hopefully help in this particular problem.

Regarding the strength of the current research, it demonstrated a high reliability level for the volumes of each anatomical mandible component with an intra-observer and inter-observer reliability of more than 0.90, as reported in Table 5. To the best of our knowledge, no previous study has attempted to determine which anatomical mandible three-dimensional component/s, i.e., the condyle, ramus, hemibody or hemimandible, is/are more affected by JIA. Moreover, this is the first study to the authors’ knowledge to use a control group matched for skeletal class and intermaxillary divergence. This particular choice was made upon the consideration of the craniofacial characteristics of JIA subjects with TMJ involvement according to Hu et al. [60], that is, a high-angle skeletal class II sagittal relationship. In fact, as already reported by Saccucci et al. [61], class II patients present significantly smaller condylar volumes compared to skeletal classes I and III. Furthermore, high-angle subjects show significantly lower condylar volumes compared to normal- and low-angle patients [62]. The authors’ choice aimed at reducing selection biases. Otherwise, it would not have been possible to understand whether the differences between JIA and non-JIA groups would be associated either with the rheumatic condition or with the most prevalent craniofacial characteristics of JIA patients (that is, high-angle class II sagittal relationship).

## Conclusions

In conclusion, the present retrospective study showed that patients with unilateral JIA have an affected hemimandible, condyle and ramus with a significantly smaller volume than those of the unaffected side. The most significant difference between the anatomical component involved in the rheumatic disease and those who have not been affected was measured at the level of the condyle.

Furthermore, the mandibular ramus volume of patients affected by JIA plays an important role in the genesis of mandibular asymmetry, causing an asymmetrical growth with a marked volumetric deficit on the affected side. There were no significant reductions among groups in the volume of the mandibular hemibody. The largest total mandibular volume was observed in the control group, followed by the unilateral JIA group and, lastly, by the bilateral JIA group.

## Data Availability

The data underlying this article will be shared on reasonable request to the corresponding author.
